# A long-term recurrence-free case of colorectal cancer with 13 simultaneous liver metastases: A case report

**DOI:** 10.1016/j.ijscr.2024.110600

**Published:** 2024-11-12

**Authors:** Masataka Nakagawa, Daisuke Sumitani, Keiso Matsubara, Hiroshi Ota, Masatsugu Yano

**Affiliations:** Department of Surgery, JR Hiroshima Hospital, 3-1-36 Hutabanosato, Higashi-ku, Hiroshima 732-0057, Japan

**Keywords:** Chemotherapy, Colorectal cancer, Liver metastases, Liver resection, Primary tumor

## Abstract

**Introduction:**

Metastatic liver tumors result from distant metastasis of a primary tumor. While chemotherapy is the treatment of choice, liver resection is aggressively performed for metastatic liver cancer derived from colorectal cancer. However, during chemotherapy, some disappearing liver metastases (DLMs) can be undetectable on computed tomography (CT), and surgical treatment remains challenging.

**Presentation of case:**

A 48-year-old woman with abdominal pain and constipation was diagnosed with multiple liver metastases of colorectal cancer (CRLM) origin after a thorough examination involving CT and ethoxybenzyl-magnetic resonance imaging. Thirteen simultaneous CRLM were observed (largest metastasis diameter, 37 mm). Resection of the primary tumor (laparoscopy-assisted left colon resection + D3 dissection) was performed. Following eight courses of chemotherapy with mFOLFOX6 + panitumumab, only two CRLM and 11 DLMs were detectable on CT. With no new lesions identified, the patient underwent anterior segment resection and segment 3 and segment 7 partial hepatectomies. Contrast-enhanced intraoperative ultrasonography was performed, and all detectable lesions were resected. However, pathology results showed three CRLM in the anterior segment and no tumor cells in the segment 3 and segment 7 specimens. Postoperatively, the patient received eight courses of adjuvant chemotherapy with capecitabine and oxaliplatin (with capecitabine as a single agent beginning mid-course). The patient is currently alive and recurrence-free 3.5 years post-hepatic resection.

**Discussion:**

The utility of EOB-MRI in the detection of DLMs has been demonstrated. The incidence of residual disease and subsequent early recurrence at sites diagnosed as DLMs on CT is reported to be approximately 80 %. Although aggressive resection of resectable DLMs is desirable to the extent that residual liver function can be preserved, recurrence is frequent and long-term careful follow-up is considered important.

**Conclusion:**

Our patient, with multiple CRLM, responded to chemotherapy and underwent conversion surgery following resection of the primary tumor. Surgeons should consider possible surgical resection and DLM management when selecting the primary treatment.

## Highlights

• We used EOB-MRI and CE-IOUS detect and successfully resect DLM in multiple CRLM.
• Detecting DLM with EOB-MRI, followed by palpation and CE-IOUS is appropriate.
• If technically possible, lesions with EOB-MRI/CE-IOUS detection should be resected.
• DLMs and lesions not detected by EOB-MRI or CE-IOUS should be followed closely.

## Abbreviations

CE-IOUS: contrast-enhanced intraoperative ultrasonography
CR: complete response
DLMs: disappearing liver metastases
EOB-MRI: Ethoxybenzyl-magnetic resonance imaging
CRLM: liver metastases of colorectal cancer
pCR: pathologic complete response

## 1 Introduction

Colorectal cancer is the third leading cause of cancer-related deaths worldwide [1]. Liver metastases of colorectal cancer (CRLM) occur in 25 %–30 % of patients, resulting in death in two-thirds of cases [1,2]. The 60-month overall survival rates in patients without and with CRLM are reported to be 60.9 % and 14.8 %, respectively [3]. In recent years, advances in chemotherapy and liver resection techniques have led to the introduction of aggressive liver resection for CRLM deriving from colorectal cancer [4]. Preoperative chemotherapy for CRLM of colorectal cancer results in tumor shrinkage in many cases; however, disappearing liver metastases (DLMs) can also be present, and their frequency is reported to range from 7 % to 37 % [5]. Moreover, most cases of DLMs are reported to recur locally within 2 years [6]. Tumor shrinkage following chemotherapy is considered a highly reliable indicator of response to chemotherapy; however, the incidence of residual disease or early recurrence at sites diagnosed as DLMs on computed tomography (CT) has been reported to be approximately 80 % [4,7]. Ethoxybenzyl-magnetic resonance imaging (EOB-MRI) and contrast-enhanced intraoperative ultrasonography (CE-IOUS) are useful for detecting DLMs with high sensitivity [8]. In this case report, we present a patient with multiple CRLM of colorectal cancer in which DLMs were detected using EOB-MRI and CE-IOUS and successfully resected. This case is reported according to the SCARE 2023 criteria [9].

## 2 Presentation of case

**Fig. 1:**
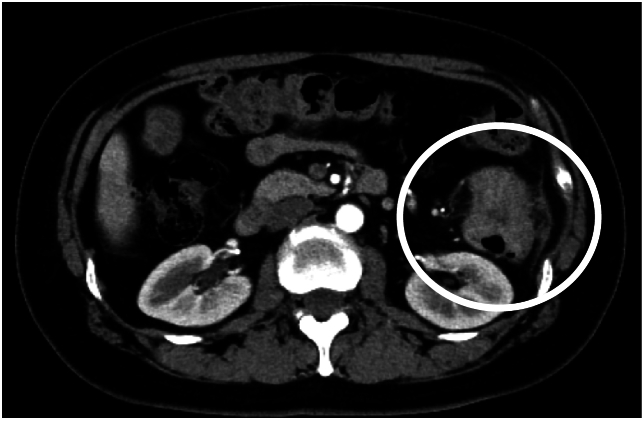
Imaging findings for the diagnosis of colorectal cancer. Computed tomography (CT) shows a 5 cm mass in the splenic curvature of the descending colon (white circles).

**Fig. 2:**
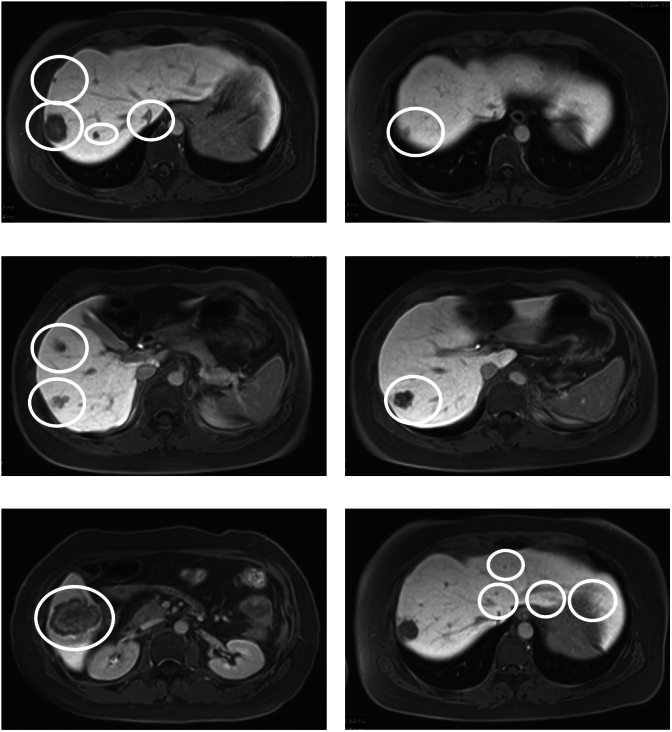
Ethoxybenzyl-magnetic resonance imaging (EOB-MRI) detected 13 masses with a maximum diameter of 37 mm in the liver (white circles).

**Fig. 3:**
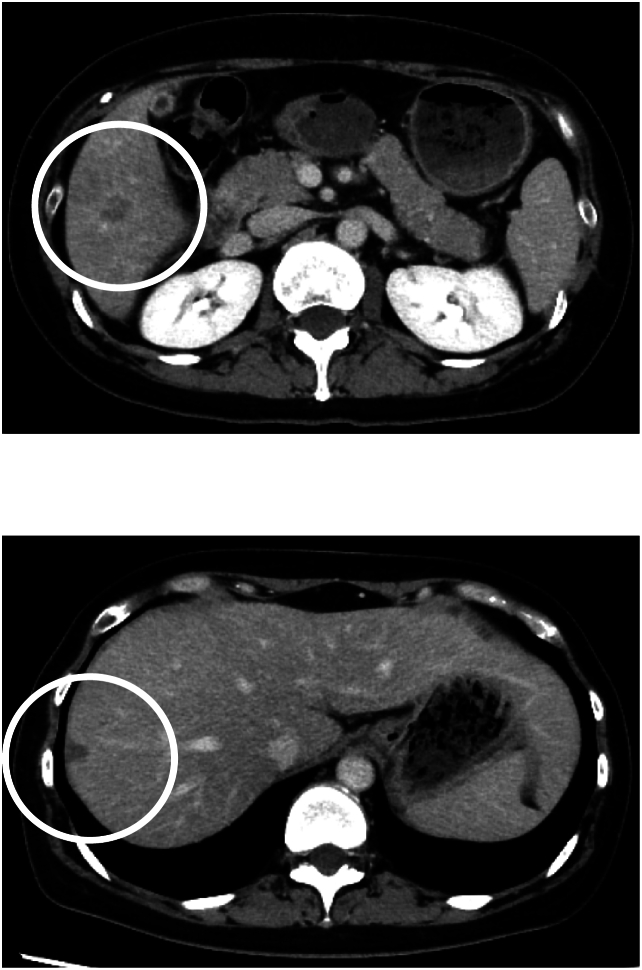
CT scan identifying two liver metastases and 11 disappearing liver metastases (white circles).

**Fig. 4:**
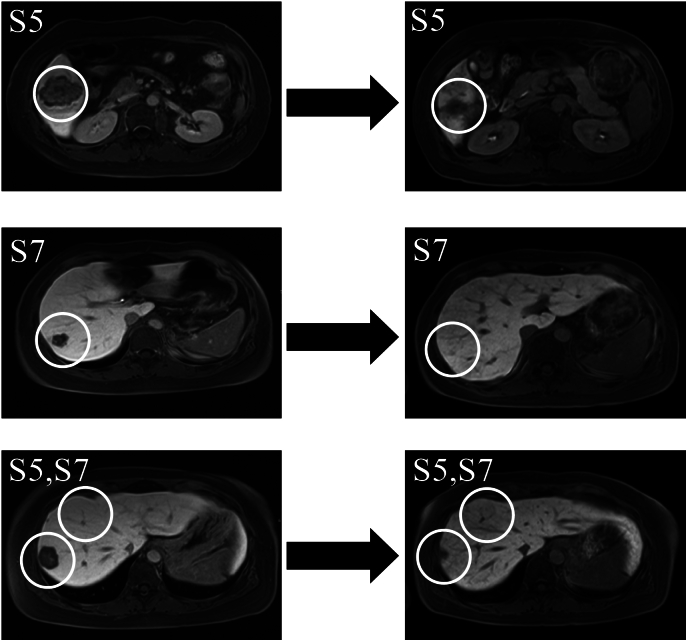
EOB-MRI shows a reduction in S5 and S7 lesions after eight courses of FOLFOX-6 (levofolinic acid, 5-fluorouracil, and oxaliplatin) (white circles). Left column, image taken prior to chemotherapy; right column, image taken post-chemotherapy.

A 48-year-old woman visited our hospital for further examination in relation to abdominal pain and constipation. Blood test results indicated abnormally low levels of hemoglobin (10.4 g/dL) and high levels of carcinoembryonic antigen (37.9 ng/mL). Colonoscopy findings indicated a type 2 tumor in the descending colon. CT scans showed a 5 cm mass in the splenic curvature of the descending colon (Fig. 1). EOB-MRI highlighted 13 masses in the liver, with a maximum diameter of 37 mm (Fig. 2). No other distant metastases were observed. The patient was diagnosed with multiple CRLM origins (T4b [SI: mural peritoneum], N1a, H2, P0, M1a[H3], and cStage IVa). Primary tumor resection (laparoscopic-assisted left colon resection + D3 dissection) was performed, and local pathology results indicated pT3N1b. The patient was discharged on postoperative day 13. Postoperative CT showed 13 CRLM. In DNA analysis to decide the chemotherapy regimen using the surgical specimen, the results of *RAS* (*KRAS* and *NRAS*) mutation and *BRAF* were negative. Because *RAS* and *BRAF* are wild-type genes, tailored adjuvant chemotherapy was used: intravenous panitumumab combined with modified FOLFOX-6 [levo-folinic acid, 5-fluorouracil, and oxaliplatin]. Eight biweekly cycles were conducted. The patient had only two CT-detectable CRLM and 11 DLMs (Fig. 3). Four CRLM were detectable on EOB-MRI: two were observed in segment 5 and two were observed in segment 7 (Fig. 4). Anterior segment resection and segment 3 and segment 7 partial hepatectomies were performed, and the segment 3 lesion, detected using CE-IOUS, was excised. Pathological examination results indicated three CRLM in the anterior segment and no tumor cells in the segment 3 and segment 7 specimens, which was considered a pathologic complete response (pCR). Postoperatively, eight courses of adjuvant chemotherapy with capecitabine and oxaliplatin (with capecitabine as a single agent beginning mid-course) were administered. Currently, the disease has not recurred.

## 3 Discussion

Chemotherapy is used to treat patients with CRLM, and it may prolong survival and render CRLM resectable [10]. Chemotherapy is also used in the neoadjuvant setting prior to surgery for resectable CRLM [11]. Some CRLM are DLMs and are not observed in imaging studies during chemotherapy. The frequency of DLMs has been reported to range from 7 % to 37 % [5]. Tumor shrinkage after chemotherapy has been reported to be a highly reliable indicator of response to chemotherapy [7]. Conditions that predispose patients to DLMs include tumor diameter < 2 cm, increased number of chemotherapy sessions, oxaliplatin-based chemotherapy, tumor diameter > 3 cm, and synchronous CRLM [12]. However, the rate of residual liver recurrence after CRLM resection is as high as 70 %, and the incidence of residual disease and early recurrence at sites diagnosed as DLMs on CT is approximately 80 % [4,7,13]. Additionally, complete response (CR) after chemotherapy based on imaging diagnosis is not equivalent to pCR. Imaging CR is largely dependent on image quality, and concordance with pCR has been reported to range from 20 % to 80 % [5]. Therefore, there is a risk of recurrence if a DLM is not resected or ablation therapy is not performed. EOB-MRI and CE-IOUS have recently been shown to be useful for detecting such DLMs with high sensitivity [10,14,15]. In comparing results concerning the recurrence of CRLM or residual tumors, EOB-MRI appears superior to CT images. After radiological examination using CT and EOB-MRI, recurrence rates were reported to range from 31 % to 33 % and from 6 % to 11 %, respectively [16]. Oba et al. reported that of 275 DLMs in 59 patients, 26 % could be identified and resected using EOB-MRI, of which 92 % had cancer cells. After additional CE-IOUS, 60 % of DLMs could be identified, of which 77 % had cancer cells; thus, 8 % of the DLMs that EOB-MRI or CE-IOUS could not identify contained cancer cells [17]. In our case, CT showed two CRLM, whereas EOB-MRI showed four, meaning that two CRLM could not be determined using CT. Pathology findings indicated three metastatic lesions in the anterior segment, and two of these lesions were diagnosed as CRLM using preoperative EOB-MRI. This indicates that EOB-MRI did not detect one CRLM and that its detection rate was not 100 %. Given the recurrence rate of residual DLMs is approximately 80 %, this would appear to support the use of maximum resection at the time of hepatic resection. However, the method of hepatic resection remains controversial. For DLMs that cannot be identified using imaging, the location of the tumor can be predicted, and the tumor can be resected by referring to the tumor location prior to chemotherapy. However, this method involves a larger area of liver resection and may be associated with increased blood loss, prolonged operative time, and increased complications. In our patient, the treatment plan was to resect three CRLM in segment 5 and one CRLM in segment 7 and to only resect other small lesions that could be observed on CE-IOUS. A wider extent of hepatectomy may reduce residual liver function and render the liver unresectable if the tumor recurs. From a retrospective perspective, partial resection of the anterior segment lesion may have been possible. However, it should be noted that CE-IOUS did not detect lesions that had not already been detected on preoperative MRI, but some lesions may have been missed during the partial resection. Therefore, in practice, it is appropriate to use preoperative EOB-MRI to detect DLMs and then carefully search for lesions by palpation and CE-IOUS. Tani et al. also proposed that, if technically feasible, all lesions detected using EOB-MRI or CE-IOUS should be resected [18]. In addition, DLMs and lesions not detected by EOB-MRI or CE-IOUS require close follow-up.

## 4 Conclusions

We present a patient with multiple CRLM of colorectal cancer that were successfully resected and treated with chemotherapy following resection of the primary tumor. It is important to consider the management of DLMs when selecting treatment.

## Research registration number

None.

## Guarantor

Masataka Nakagawa, Daisuke Sumitani, Keiso Matsubara, Hiroshi Ota, Masatsugu Yano

## CRediT authorship contribution statement

MN drafted the manuscript. DS supervised drafting of the manuscript. KM, HO and MY performed the surgical procedure and perioperative management. All the authors have read and approved the final version of the manuscript.

## Ethical approval

The ethics committee of our institution approved all procedures in this case.

## Consent

Written informed consent was obtained from the patient for the publication of the case and any accompanying images. A copy of the written consent is available for review by the Editor-in-Chief on request.

## Sources of funding

No funding.

## Declaration of competing interest

The authors declare that they have no competing interest.
